# Supplementary material for: Within-individual variation of HbA1c measurements in primary care: A retrospective cohort study
Source: PLoS One. 2025 Oct 3;20(10):e0333438. doi: 10.1371/journal.pone.0333438 (PMC12494249; doi:10.1371/journal.pone.0333438)
Supplement: S1 File — (DOCX) [file pone.0333438.s001.docx]

# Supplementary Materials

## Subgroup analysis

Table S1 Other subgroup analyses.

|  |  | N | (%) | Mean (mmol/mol) | CV | (95%CI) |
| --- | --- | --- | --- | --- | --- | --- |
| Comorbidities | No comorbidity (incl DM) | 36,157 | (6.2) | 37.52 | 0.102 | (0.101 - 0.102) |
|  | PDM, DM1 or DM2 with no comorbidity | 66,710 | (11.4) | 61.71 | 0.189 | (0.188 - 0.189) |
|  | Hypertension | 324,047 | (55.2) | 52.60 | 0.194 | (0.194 - 0.195) |
|  | Asthma | 102,976 | (17.5) | 52.38 | 0.203 | (0.203 - 0.204) |
|  | COPD | 47,079 | (8.0) | 52.31 | 0.200 | (0.199 - 0.200) |
|  | Hyperthyroidism | 12,167 | (2.1) | 51.30 | 0.193 | (0.192 - 0.194) |
|  | Hypothyroidism | 65,616 | (11.2) | 51.81 | 0.195 | (0.194 - 0.196) |
|  | CKDstage3 to 5 | 97,611 | (16.6) | 53.64 | 0.197 | (0.196 - 0.197) |
|  | Heart failure | 22,740 | (3.9) | 55.51 | 0.206 | (0.205 - 0.207) |
|  | Heart disease | 102,420 | (17.4) | 53.01 | 0.190 | (0.189 - 0.190) |
|  | Ischaemic stroke | 12,453 | (2.1) | 53.70 | 0.199 | (0.198 - 0.200) |
|  | Haem. Stroke | 3,926 | (0.7) | 52.06 | 0.204 | (0.201 - 0.206) |
|  | Cancer | 50,146 | (8.5) | 51.91 | 0.190 | (0.189 - 0.190) |
|  | PDM, DM1 or DM2 with 1 or more comorbidity | 255,798 | (43.6) | 57.68 | 0.185 | (0.184 - 0.185) |
|  | 1 or more comorbidity with no PDM, DM1 or DM2 | 80,444 | (13.7) | 38.55 | 0.087 | (0.086 - 0.087) |
| Diabetic medication* | None | 253,534 | (43.2) | 40.47 | 0.077 | (0.077 - 0.077) |
|  | Diabetic drug but not insulin | 207,239 | (35.3) | 57.39 | 0.184 | (0.184 - 0.184) |
|  | Insulin | 81,420 | (13.9) | 69.66 | 0.170 | (0.170 - 0.170) |
|  | All diabetic drugs | 299,000 | (50.9) | 61.39 | 0.190 | (0.190 - 0.190) |
| Region | East Midlands | 3,815 | (0.6) | 59.04 | 0.164 | (0.162 - 0.166) |
|  | East of England | 17,745 | (3.0) | 54.48 | 0.186 | (0.185 - 0.187) |
|  | London | 62,903 | (10.7) | 51.29 | 0.196 | (0.196 - 0.197) |
|  | North East | 7,255 | (1.2) | 55.31 | 0.184 | (0.182 - 0.185) |
|  | North West | 87,175 | (14.9) | 47.55 | 0.188 | (0.188 - 0.189) |
|  | Northern Ireland | 38,782 | (6.6) | 50.36 | 0.209 | (0.208 - 0.210) |
|  | Scotland | 87,135 | (14.8) | 57.68 | 0.206 | (0.206 - 0.207) |
|  | South Central | 31,671 | (5.4) | 57.84 | 0.192 | (0.192 - 0.193) |
|  | South East Coast | 49,530 | (8.4) | 53.38 | 0.198 | (0.198 - 0.199) |
|  | South West | 31,404 | (5.3) | 54.42 | 0.191 | (0.190 - 0.192) |
|  | Wales | 111,820 | (19.0) | 50.25 | 0.204 | (0.203 - 0.204) |
|  | West Midlands | 53,147 | (9.1) | 50.67 | 0.183 | (0.182 - 0.183) |
|  | Yorkshire & Humber | 4,641 | (0.8) | 55.72 | 0.190 | (0.188 - 0.192) |
| Alcohol consumption | Current drinker | 344,894 | (58.8) | 51.73 | 0.197 | (0.197 - 0.198) |
|  | Ex drinker | 10,562 | (1.8) | 54.68 | 0.218 | (0.217 - 0.220) |
|  | Teetotal | 119,829 | (20.4) | 53.53 | 0.202 | (0.201 - 0.202) |
|  | Missing | 111,738 | (19.0) | 52.57 | 0.206 | (0.205 - 0.206) |
| Smoker Status | Current Smoker | 117,878 | (20.1) | 53.58 | 0.214 | (0.213 - 0.214) |
|  | Ex-smoker | 127,989 | (21.8) | 52.63 | 0.196 | (0.196 - 0.197) |
|  | Never smoked | 278,076 | (47.4) | 51.72 | 0.195 | (0.194 - 0.195) |
|  | Missing | 63,080 | (10.7) | 51.9 | 0.206 | (0.205 - 0.206) |

Note that for diabetic medication, the total for N does not equal the total participants in the study (578,023) since participants that had a change in medication status but had less than four measurements before or after the change were excluded. The denominator of 578023 has been used for the percentage calculation as it represents the total participants included.


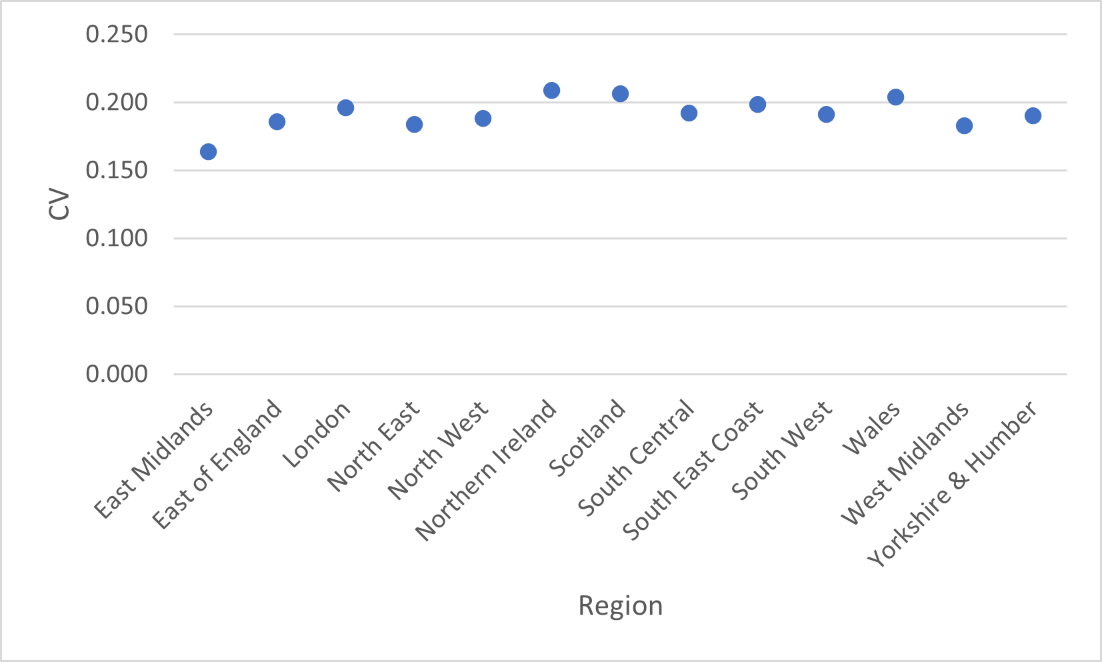


Figure S1 CVT by region


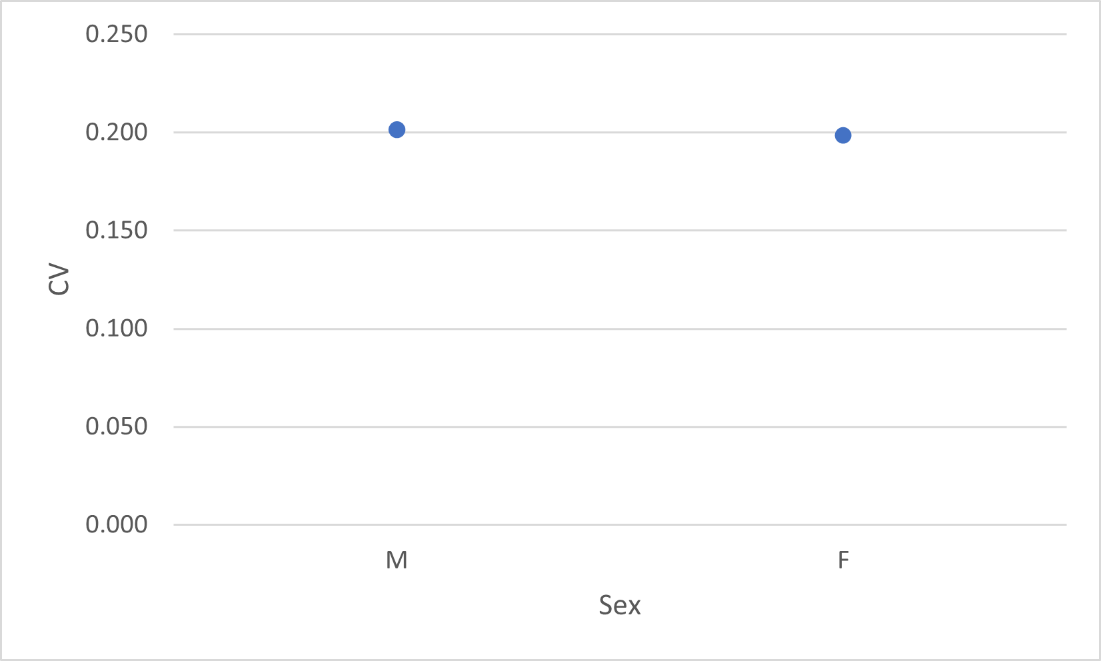


Figure S2 CVT by sex


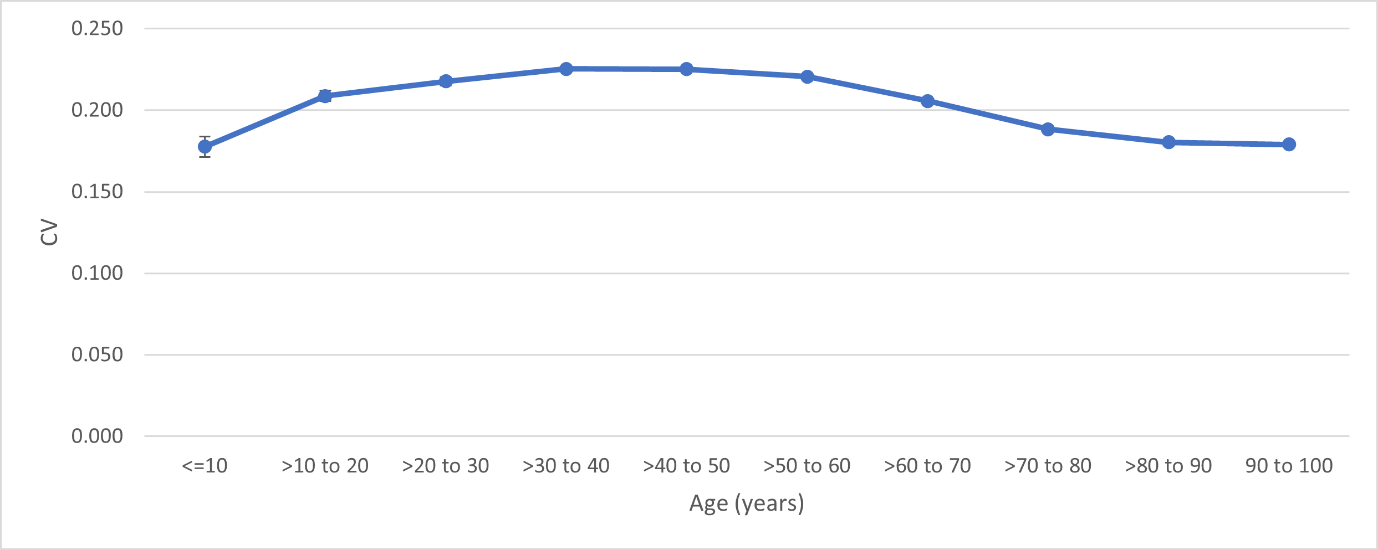


Figure S3 CVT by age


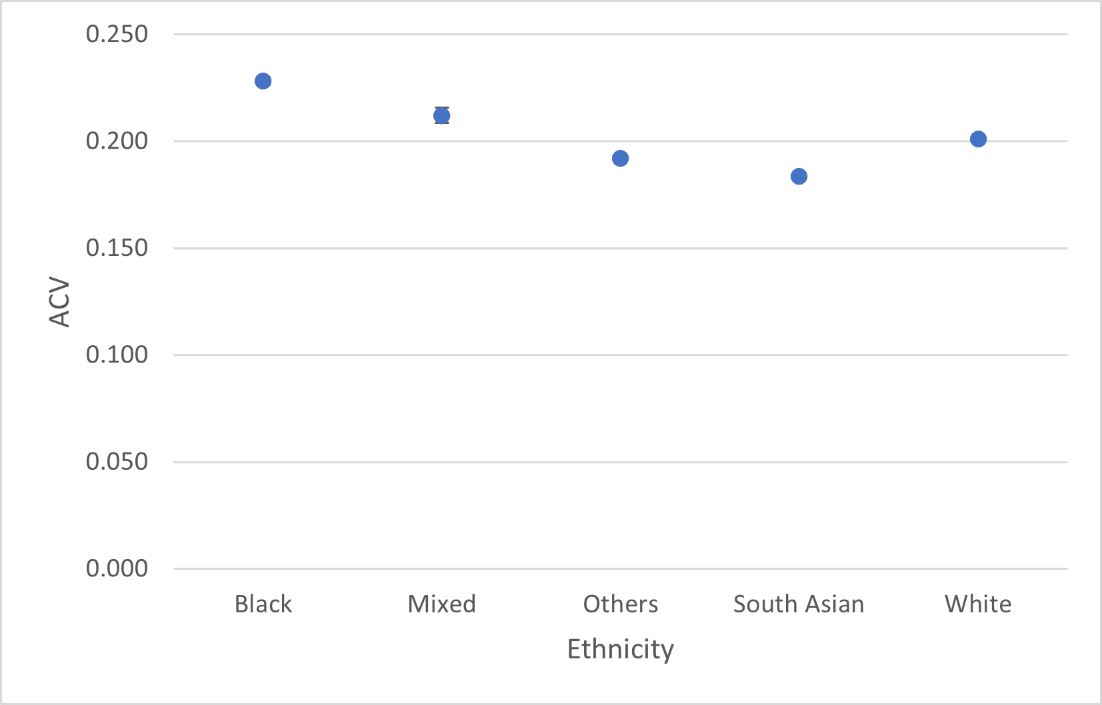


Figure S4 CVT by ethnicity


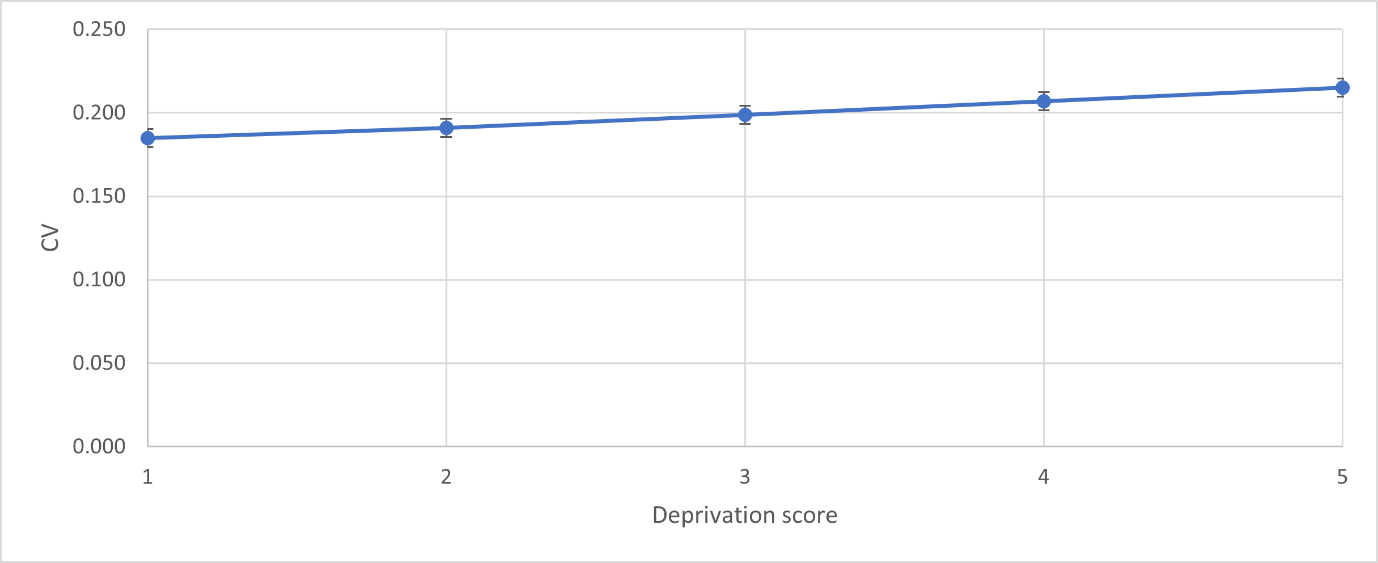


Figure S5 CVT by Townsend deprivation score


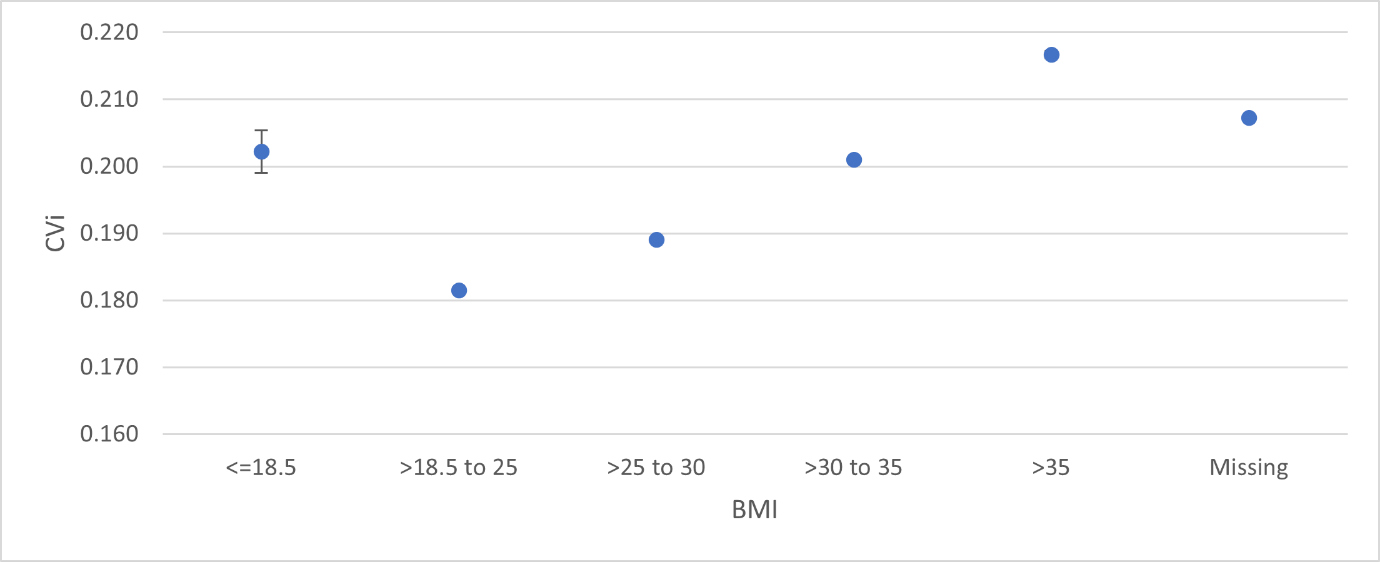


Figure S6 CVT by BMI


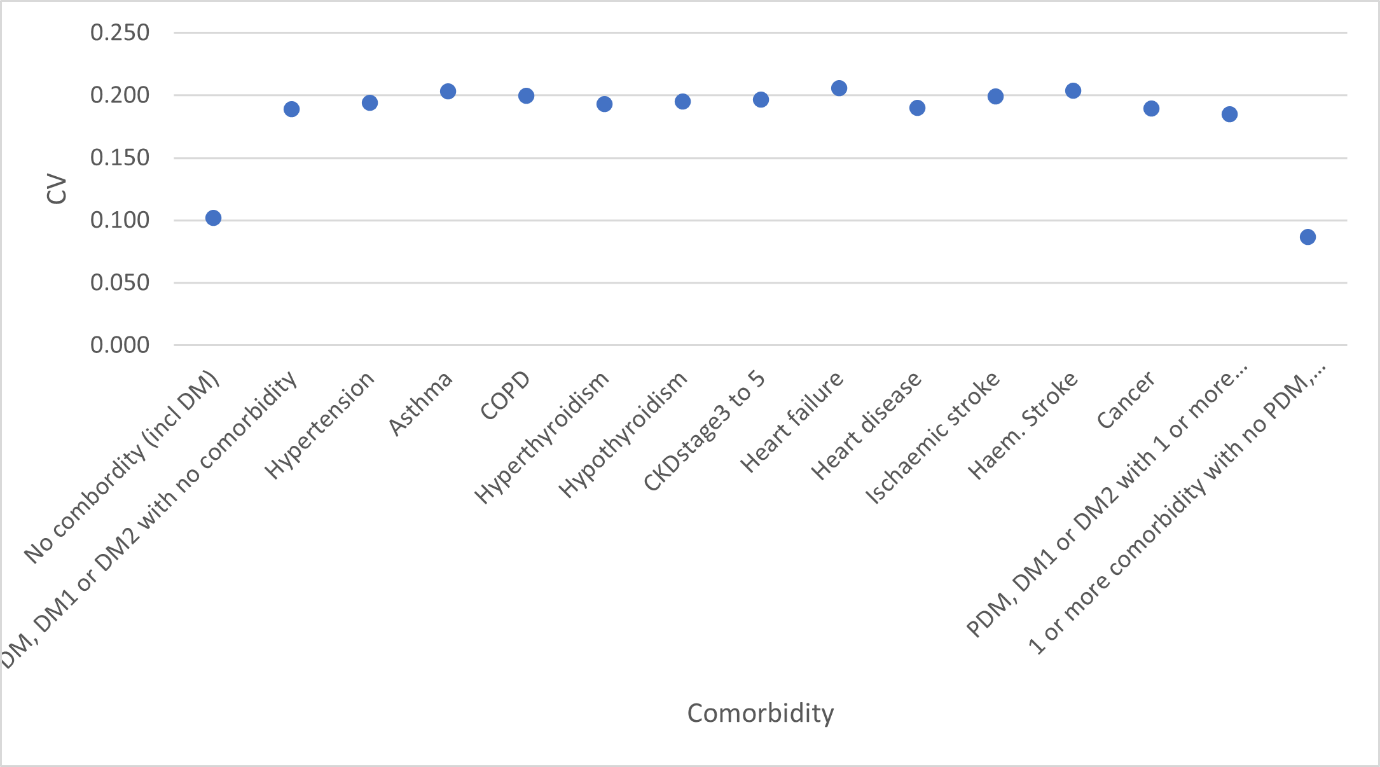


Figure S7 CVT by comorbidities


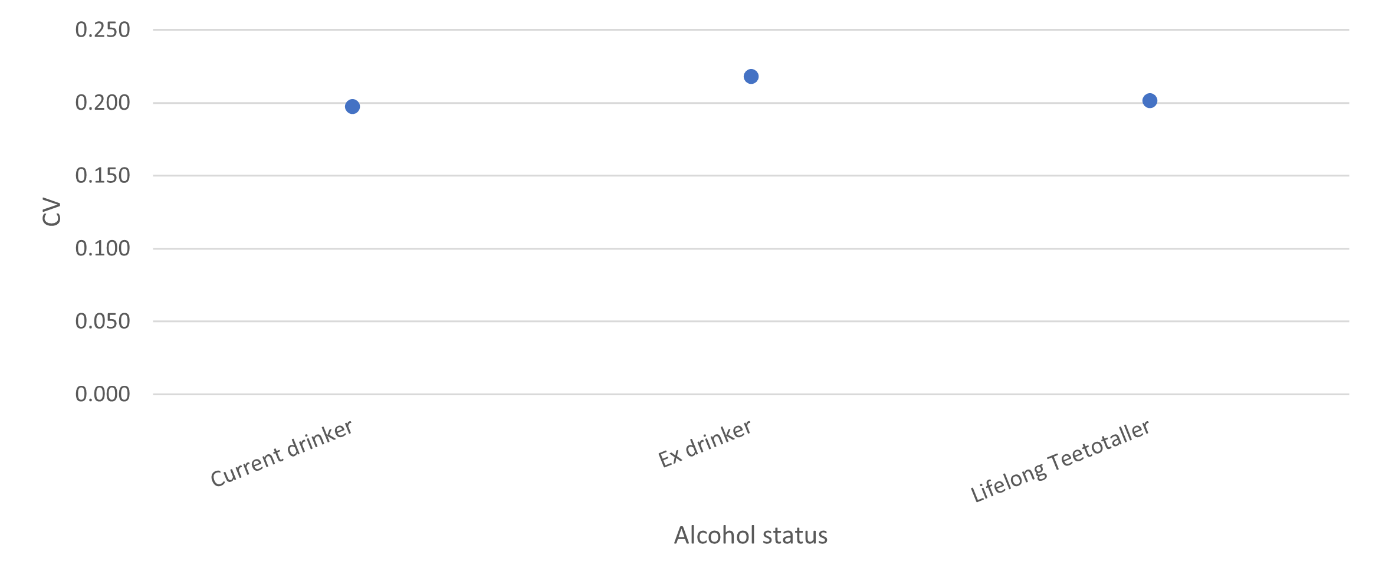


Figure S8 CVT by alcohol consumption


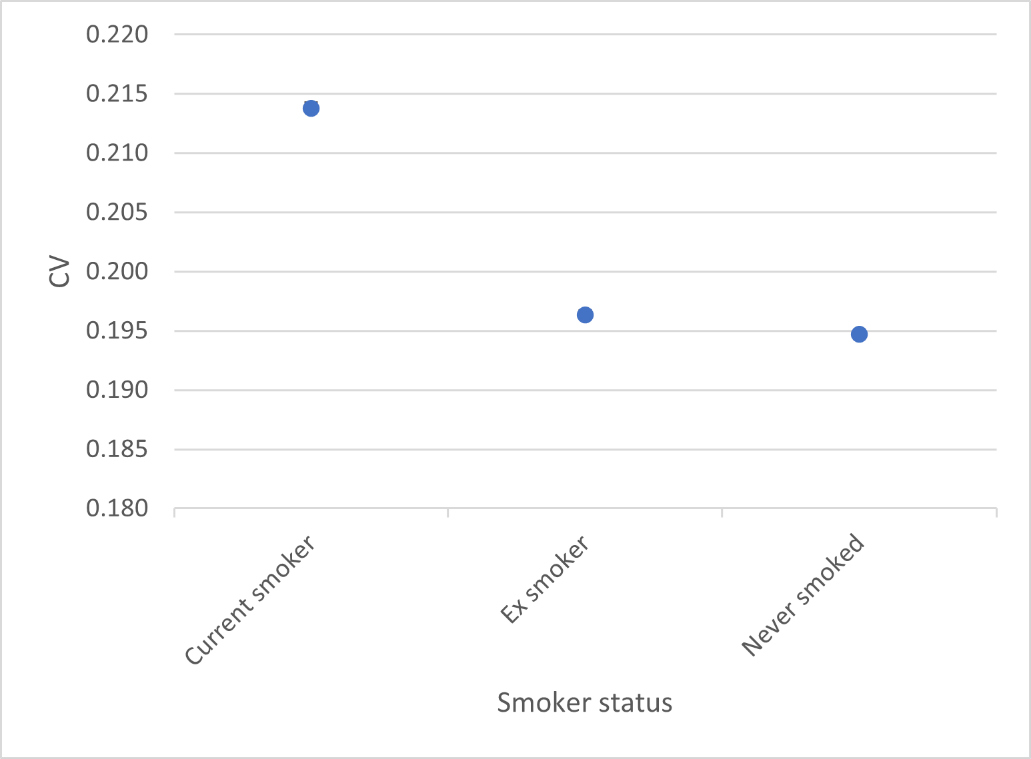


Figure S9 CVT by smoker status

##

## Sensitivity analyses

Table S2 Sensitivity analyses. Note that the total participants for year is much lower than the total participants in the study (587,023) because only participants with four measurements *in the same year* were included for this calculation.

|  |  | N | % | Mean | CV (95%CI) | 95% LCI | 95% UCI |
| --- | --- | --- | --- | --- | --- | --- | --- |
| Year | 2010 | 7,300 | 1.2 | 64.92 | 0.158 | 0.157 | 0.160 |
|  | 2011 | 8,963 | 1.5 | 66.31 | 0.162 | 0.161 | 0.164 |
|  | 2012 | 10,972 | 1.9 | 67.53 | 0.159 | 0.158 | 0.160 |
|  | 2013 | 13,351 | 2.3 | 66.48 | 0.161 | 0.160 | 0.162 |
|  | 2014 | 16,725 | 2.8 | 65.27 | 0.154 | 0.153 | 0.155 |
|  | 2015 | 16,738 | 2.9 | 65.88 | 0.156 | 0.155 | 0.157 |
|  | 2016 | 16,310 | 2.8 | 64.88 | 0.151 | 0.150 | 0.152 |
|  | 2017 | 15,225 | 2.6 | 64.47 | 0.153 | 0.152 | 0.154 |
|  | 2018 | 14,218 | 2.4 | 64.18 | 0.153 | 0.152 | 0.154 |
|  | 2019 | 10,188 | 1.7 | 65.01 | 0.159 | 0.157 | 0.160 |
| Number of measurements | 2 | 310,643 | N/A | 39.75 | 0.121 | 0.121 | 0.122 |
|  | 3 | 194,601 | N/A | 41.92 | 0.138 | 0.138 | 0.139 |
|  | 4 | 123,417 | N/A | 44.25 | 0.153 | 0.153 | 0.153 |
|  | 5 | 83,459 | N/A | 46.80 | 0.167 | 0.166 | 0.167 |
|  | 6 | 61,485 | N/A | 49.27 | 0.176 | 0.175 | 0.176 |
|  | 7 | 47,972 | N/A | 51.24 | 0.180 | 0.179 | 0.181 |
|  | 8 | 38,751 | N/A | 53.18 | 0.184 | 0.183 | 0.185 |
|  | 9 | 31,460 | N/A | 55.06 | 0.187 | 0.186 | 0.188 |
|  | 10 to 15 | 118,418 | N/A | 57.78 | 0.190 | 0.189 | 0.190 |
|  | 16 to 20 | 46,905 | N/A | 60.58 | 0.189 | 0.189 | 0.190 |
|  | 21 to 25 | 22,951 | N/A | 62.60 | 0.189 | 0.188 | 0.189 |
|  | 26 to 30 | 8,487 | N/A | 65.21 | 0.188 | 0.187 | 0.189 |
|  | 31 to 40 | 3,990 | N/A | 67.94 | 0.187 | 0.186 | 0.189 |
|  | 41 to 50 | 503 | N/A | 69.16 | 0.188 | 0.184 | 0.191 |
|  | 51 to 60 | 63 | N/A | 68.23 | 0.207 | 0.197 | 0.217 |
| Patient median HbA1c (%) | <=5 | 6,853 | 1.2 | 29.86 | 0.128 | 0.127 | 0.129 |
|  | >5 to 6 | 187,834 | 32.0 | 38.49 | 0.113 | 0.113 | 0.114 |
|  | >6 to 7 | 175,186 | 29.8 | 48.42 | 0.166 | 0.166 | 0.167 |
|  | >7 to 8 | 114,466 | 19.5 | 59.24 | 0.187 | 0.186 | 0.187 |
|  | >8 to <9 | 55,352 | 9.4 | 69.69 | 0.193 | 0.192 | 0.193 |
|  | >9 to 10 | 26,384 | 4.5 | 79.65 | 0.193 | 0.193 | 0.194 |
|  | >10 to 11 | 12,616 | 2.1 | 89.33 | 0.191 | 0.191 | 0.192 |
|  | >11 to 12 | 5,368 | 0.9 | 98.99 | 0.191 | 0.190 | 0.192 |
|  | >12 to 13 | 2,010 | 0.3 | 108.42 | 0.191 | 0.189 | 0.193 |
|  | >13 to 14 | 699 | 0.1 | 118.26 | 0.196 | 0.192 | 0.200 |
|  | >14 to 15 | 188 | 0.0 | 126.27 | 0.203 | 0.195 | 0.210 |
| Implausible values (%) | >=3 and <20 | 586,251 | N/A | 52.21 | 0.213 | 0.213 | 0.213 |
|  | >=4 and < 20 | 585,319 | N/A | 52.32 | 0.200 | 0.200 | 0.200 |
|  | All values < 100 | 589,255 | N/A | 51.79 | 0.240 | 0.240 | 0.241 |
|  | >=4 and <15 | 584,902 | N/A | 52.22 | 0.195 | 0.195 | 0.195 |
|  | >=4 and <20 | 585,319 | N/A | 52.32 | 0.200 | 0.200 | 0.200 |
|  | >=4 and <25 | 585,319 | N/A | 52.33 | 0.200 | 0.200 | 0.201 |
|  | >=4 and <30 | 585,319 | N/A | 52.33 | 0.200 | 0.200 | 0.201 |
| Days between measurements | < 30 | 1,263 | 0.22 | 56.24 | 0.233 | 0.227 | 0.238 |
|  | 30 to < 60 | 4,505 | 0.77 | 61.59 | 0.243 | 0.240 | 0.245 |
|  | 60 to < 90 | 14,849 | 2.53 | 63.25 | 0.222 | 0.221 | 0.223 |
|  | 90 to < 120 | 45,042 | 7.67 | 62.91 | 0.205 | 0.204 | 0.205 |
|  | 120 to < 150 | 55,321 | 9.42 | 60.85 | 0.201 | 0.201 | 0.202 |
|  | 150 to < 180 | 74,857 | 12.75 | 57.39 | 0.191 | 0.190 | 0.191 |
|  | 180 to < 210 | 87,166 | 14.85 | 54.51 | 0.178 | 0.177 | 0.178 |
|  | 210 to < 240 | 46,658 | 7.95 | 53.24 | 0.187 | 0.186 | 0.187 |
|  | 240 to < 270 | 35,549 | 6.06 | 51.17 | 0.186 | 0.185 | 0.186 |
|  | 270 to < 300 | 31,410 | 5.35 | 48.82 | 0.181 | 0.181 | 0.182 |
|  | 300 to < 330 | 30,924 | 5.27 | 46.91 | 0.171 | 0.170 | 0.172 |
|  | 330 to < 365 | 45,761 | 7.80 | 44.74 | 0.152 | 0.152 | 0.153 |
|  | 365 to < 455 | 63,605 | 10.84 | 43.04 | 0.144 | 0.144 | 0.145 |
|  | 455 to < 545 | 23,230 | 3.96 | 41.35 | 0.138 | 0.137 | 0.139 |
|  | 545 to < 635 | 11,790 | 2.01 | 40.63 | 0.133 | 0.131 | 0.134 |
|  | 635 to < 730 | 7,026 | 1.20 | 39.86 | 0.129 | 0.128 | 0.131 |
|  | more than 730 | 8,067 | 1.37 | 39.88 | 0.122 | 0.120 | 0.123 |
| Method | Linear regression | 587,023 | 100 | 52.31 | 0.200 | 0.200 | 0.201 |
|  | Arithmetic | 587,023 | 100 | 55.9 | 0.131 | 0.001* | 7.966* |
| Unit | coded as % | 344,382 | N/A | 7.42% | 0.143 | 0.143 | 0.144 |
|  | coded as mmol/mol and transformed to % | 587,023 | 100 | 6.94% | 0.138 | 0.138 | 0.138 |
|  | coded as mmol/mol and not transformed | 587,023 | 100 | 52.31 | 0.200 | 0.200 | 0.201 |

Mean is in mmol/mol unless otherwise stated. *=range.


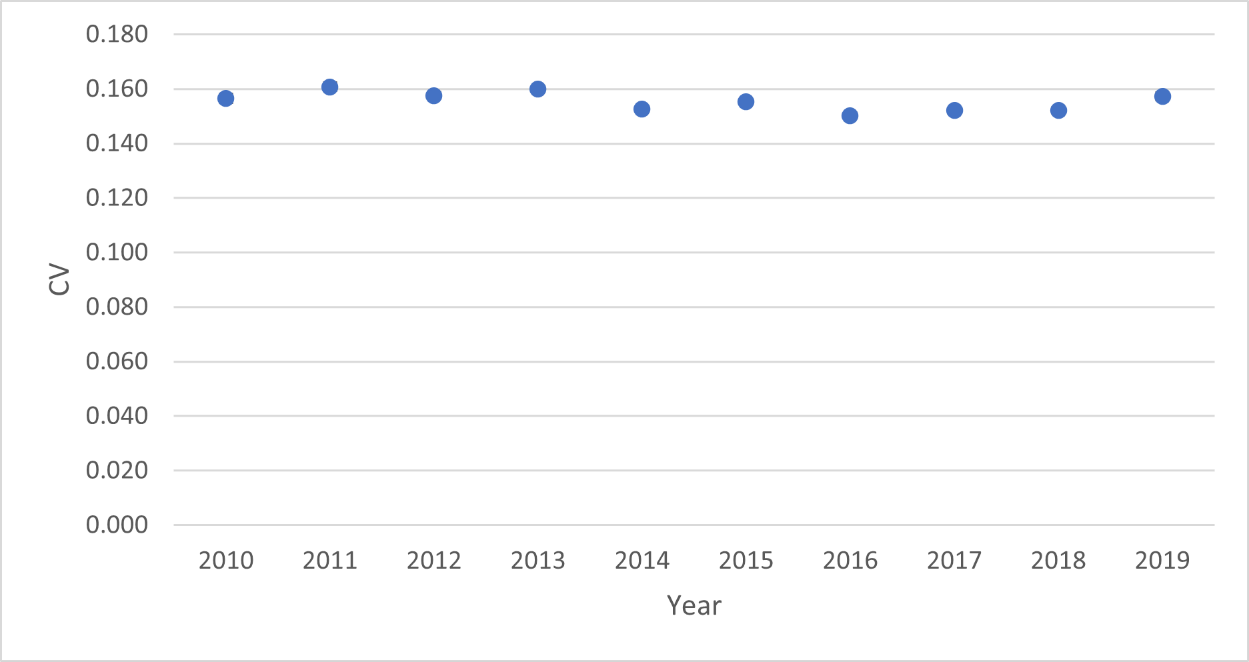


Figure S10 CVT by year


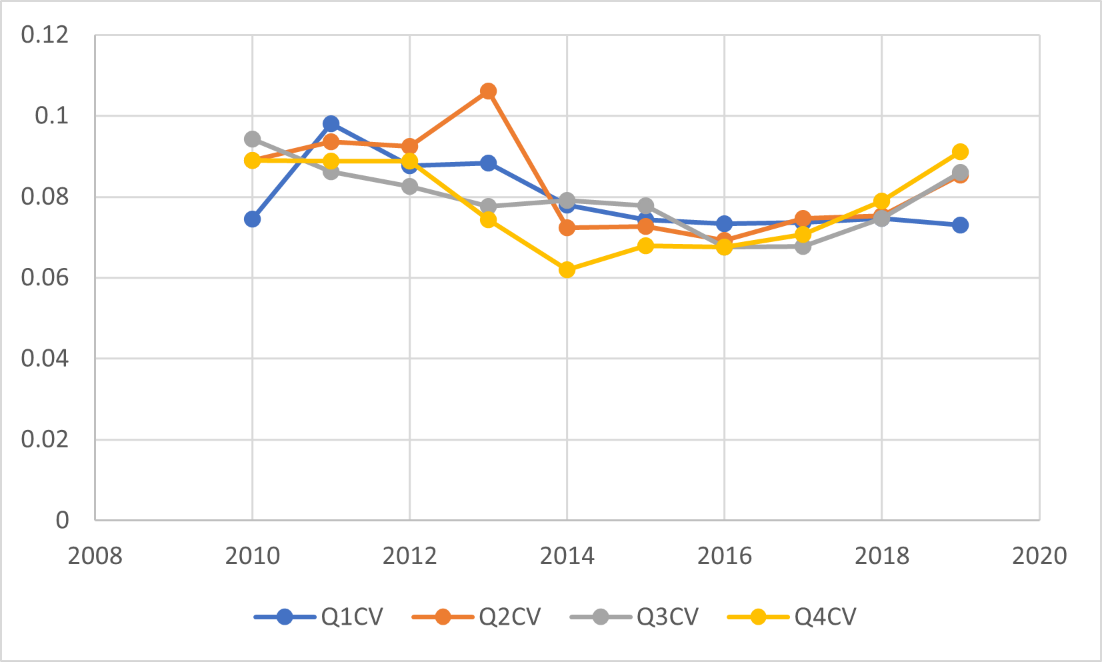


Figure S11 CVT by quarter

Table S3 CVT by quarter

| Year |  | Q1 | Q2 | Q3 | Q4 |
| --- | --- | --- | --- | --- | --- |
| 2010 |  | 0.099 | 0.120 | 0.128 | 0.120 |
| 2011 |  | 0.133 | 0.124 | 0.116 | 0.120 |
| 2012 |  | 0.115 | 0.107 | 0.112 | 0.120 |
| 2013 |  | 0.118 | 0.143 | 0.107 | 0.101 |
| 2014 |  | 0.106 | 0.096 | 0.109 | 0.085 |
| 2015 |  | 0.100 | 0.100 | 0.105 | 0.093 |
| 2016 |  | 0.099 | 0.094 | 0.092 | 0.092 |
| 2017 |  | 0.100 | 0.101 | 0.093 | 0.094 |
| 2018 |  | 0.100 | 0.103 | 0.104 | 0.107 |
| 2019 |  | 0.101 | 0.116 | 0.118 | 0.123 |


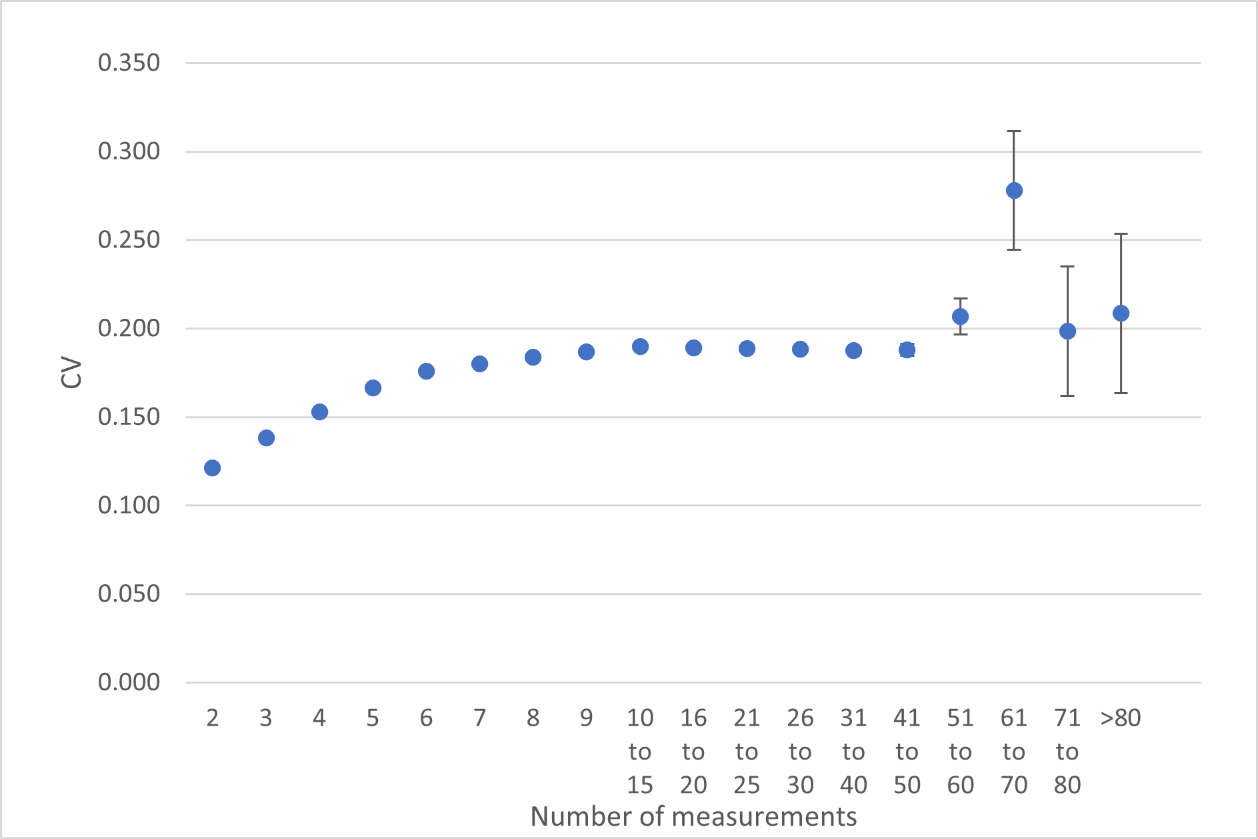


Figure S12 CVT by Number of measurements


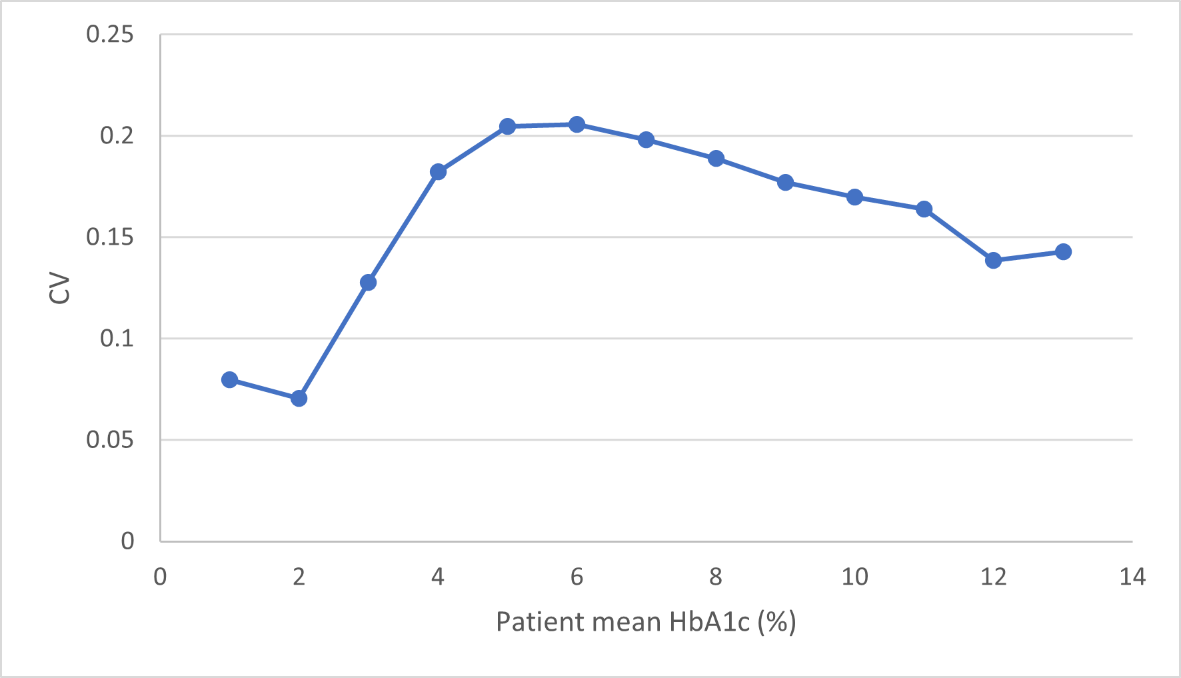


Figure S13 CVT by Mean HbA1c (%)


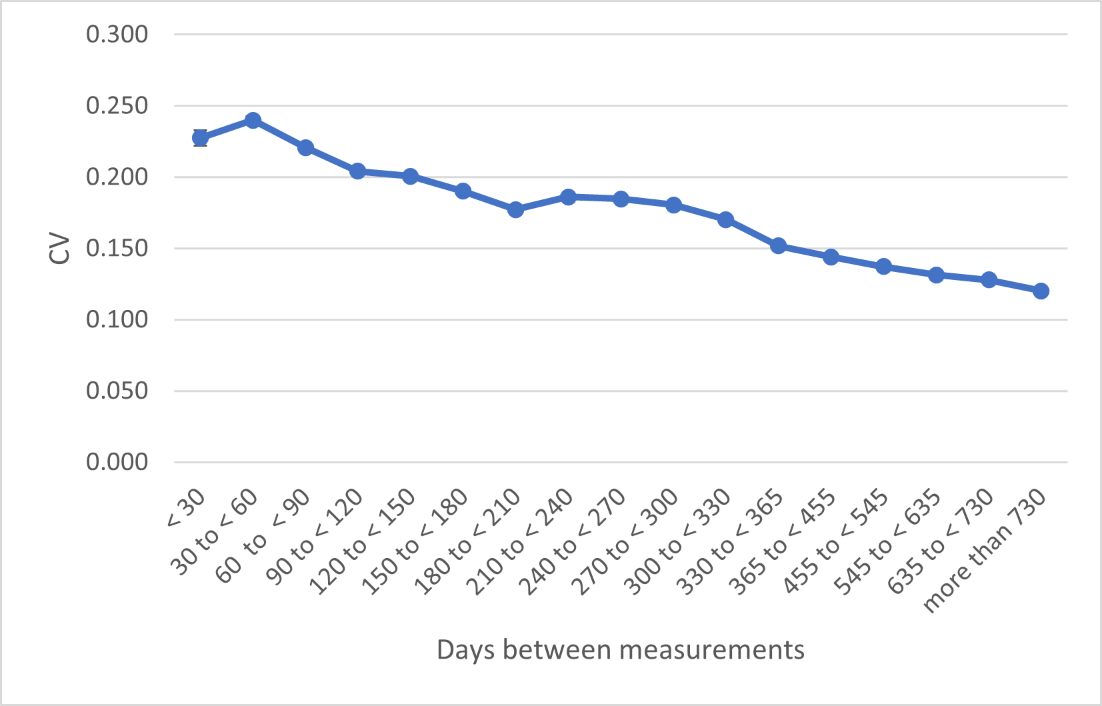


Figure S14 CVT by days between measurements


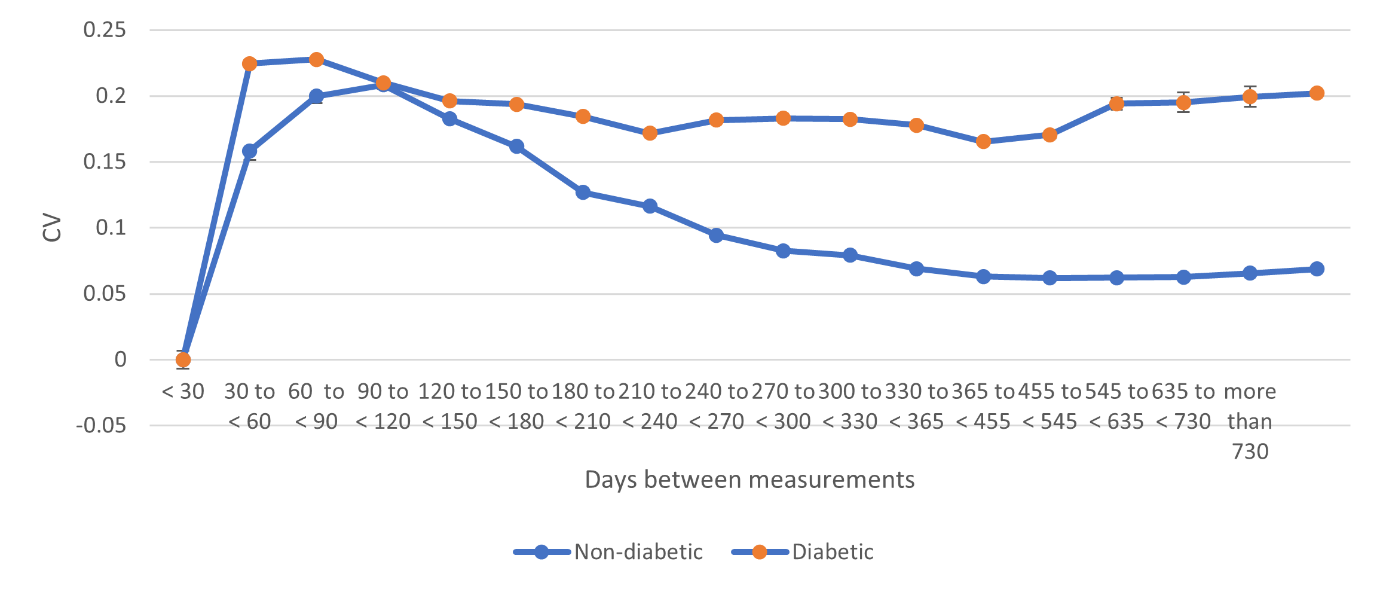


Figure S15 CVT by Days between measurements stratified by diabetic status


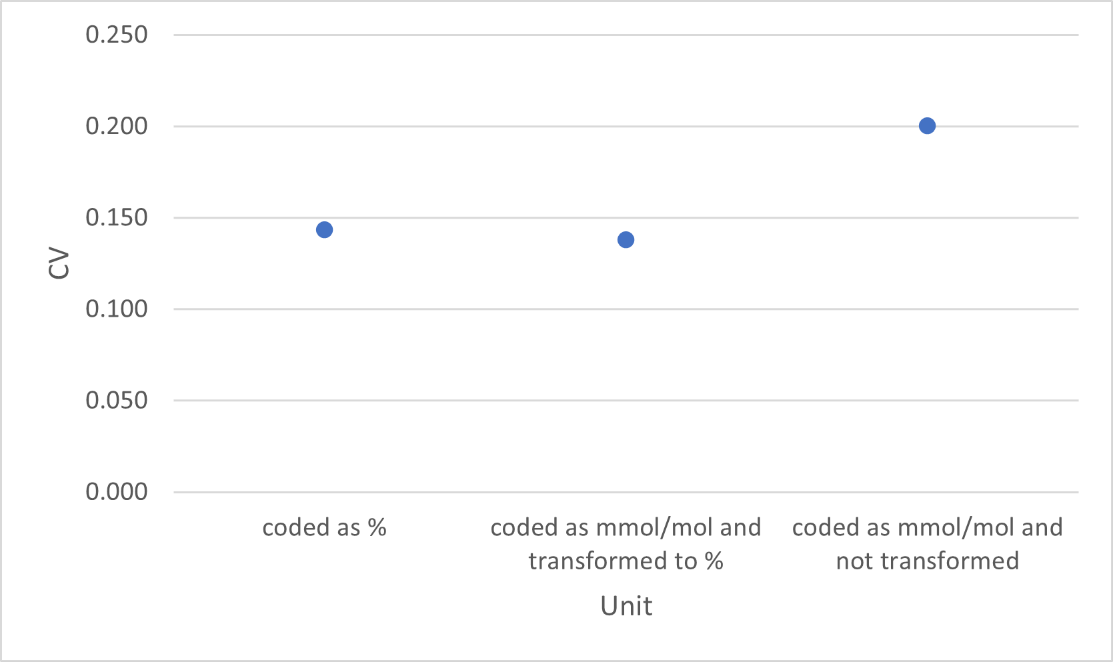


Figure S16 CVT by unit of measurement


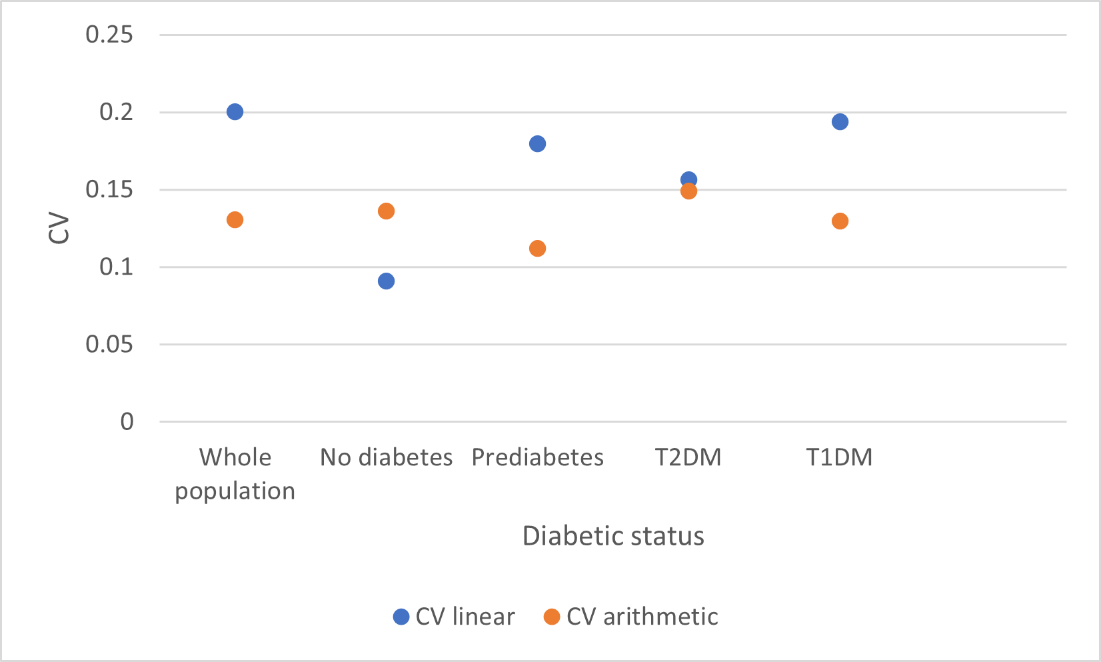


Figure S17 CVT by arithmetic calculation methods


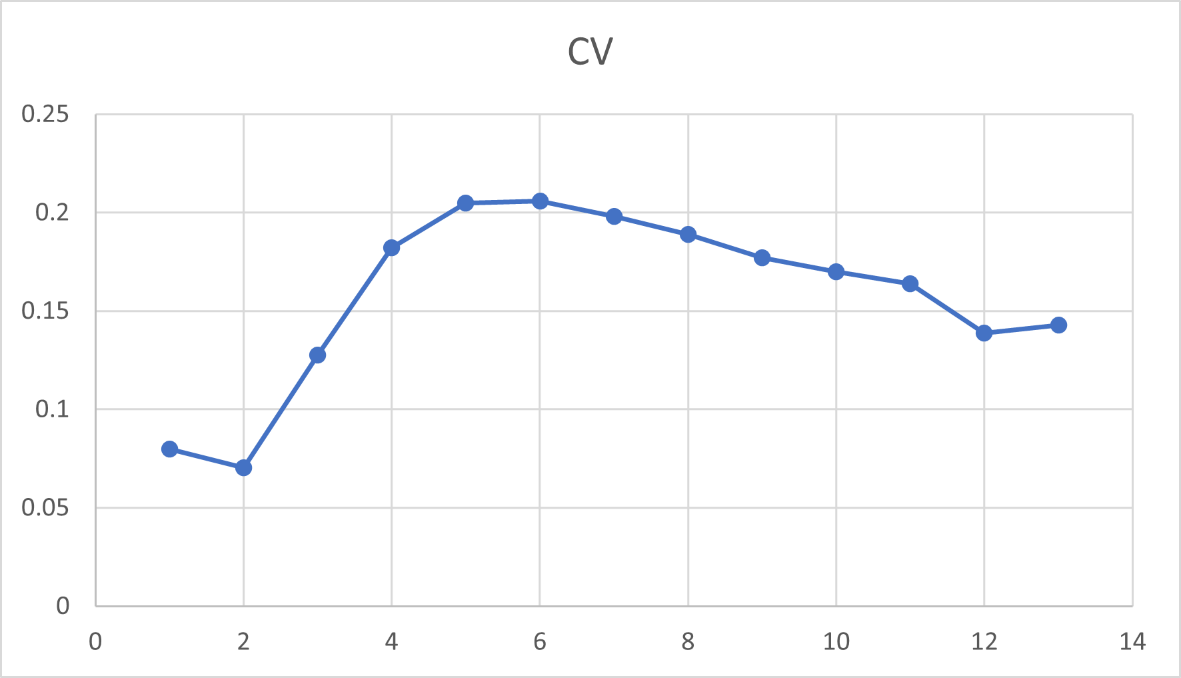


Figure S18 CVT by mean HbA1c (%)


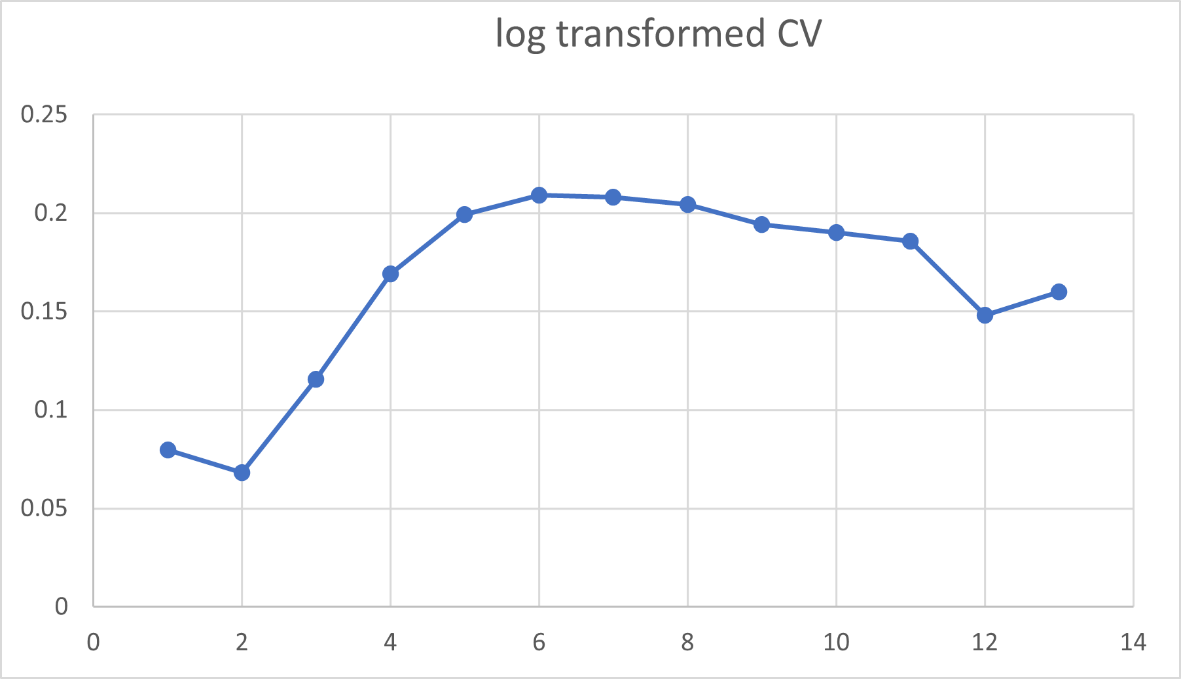


Figure S19 CVT by mean HbA1c (log-transformed data)
